# A Rationale for Schistosomiasis Control in Elementary Schools of the Rainforest Zone of Pernambuco, Brazil

**DOI:** 10.1371/journal.pntd.0000395

**Published:** 2009-03-17

**Authors:** Tereza C. Favre, Ana P. B. Pereira, Aline F. Galvão, Luciana C. Zani, Constança S. Barbosa, Otávio S. Pieri

**Affiliations:** 1 Laboratório de Ecoepidemiologia e Controle da Esquistossomose e Geohelmintoses, Instituto Oswaldo Cruz, Fundação Oswaldo Cruz, Rio de Janeiro, Brazil; 2 Departamento de Parasitologia, Instituto Aggeu Magalhães, Fundação Oswaldo Cruz, Pernambuco, Brazil; Fundação Oswaldo Cruz, Brazil

## Abstract

**Background:**

Since its beginning in 1999, the Schistosomiasis Control Program within the Unified Health System (PCE-SUS) has registered a cumulative coverage of just 20% of the population from the Rainforest Zone of Pernambuco (ZMP), northeast Brazil. This jeopardizes the accomplishment of the minimum goal of the Fifty-Fourth World Health Assembly, resolution WHA54.19, of providing treatment for schistosomiasis and soil-transmitted helminthiases (STH) to 75% of school-aged children at risk, which requires attending at least 166,000 residents in the 7–14 age range by year 2010 in that important endemic area. In the present study, secondary demographic and parasitological data from a representative municipality of the ZMP are analyzed to provide evidence that the current, community-based approach to control schistosomiasis and STH is unlikely to attain the WHA-54.19 minimum goal and to suggest that school-based control actions are also needed.

**Methodology/Principal Findings:**

Data available on the PCE-SUS activities related to diagnosis and treatment of the population from the study municipality were obtained from the State Secretary of Health of Pernambuco (SES/PE) for 2002–2006, complemented by the Municipal Secretary of Health (SMS) for 2003–2004. Data from a school-based stool survey carried out by the Schistosomiasis Reference Service of the Oswaldo Cruz Foundation (SRE/Fiocruz) in 2004 were used to provide information on infection status variation among school-aged children (7–14 years). According to the SES, from 2004 to 2006, only 2,977 (19.5%) of the estimated 15,288 residents of all ages were examined, of which 396 (13.3%) were positive for *Schistosoma mansoni*. Among these, only 180 (45.5%) were treated. According to the SMS, of the 1,766 examined in the 2003–2004 population stool survey 570 (32.3%) were children aged 7–14 years. One year later, the SRE/Fiocruz school survey revealed that the infection status among those children remained unchanged at 14%–15% prevalence. By 2006, the school-aged population was estimated at 2,981, of which 2,007 (67.3%) were enrolled as pupils.

**Conclusions:**

It is suggested that in the most troubled municipalities individual diagnosis and treatment should be concentrated in school-aged children rather than the whole population. School-based actions involving teachers and children's families may help the health teams to scale up control actions in order to attain the WHA-54.19 minimum goal. This strategy should involve health and education organs and include both enrolled and non-enrolled children.

## Author Summary

In 2001, a World Health Assembly resolution urged member states to ensure treatment against schistosomiasis and soil-transmitted helminthiasis in endemic areas with the goal of attaining a minimum target of at least 75% of all school-aged children by 2010. In the highly endemic Rainforest Zone of Pernambuco (ZMP), northeast Brazil, the Schistosomiasis Control Program has registered a cumulative coverage of only 20% of the population at risk, which jeopardizes the accomplishment of the minimum target for that area. Demographic and parasitological data from a representative municipality of the ZMP provide evidence that the current, community-based approach to control can be complemented with school-based actions. In the most troubled municipalities, individual diagnosis and treatment could be focused on school-aged children rather than whole populations without compromising the principles of the primary health care system. Local health and education teams should be encouraged to include school-based interventions to scale up coverage and achieve a rapid impact on infection.

## Introduction

Fifty-fourth World Health Assembly, resolution WHA54.19 (WHA-54.19) [1], held in May 2001, recommended Country Members to develop sustainable control activities, ensure access to medication, promote preventive measures and secure resources for the control of schistosomiasis and soil-transmitted helminthiases (STH). WHA's minimum target is to provide coverage to 75% of all school-aged children at risk by 2010 [1].

In Brazil, the Ministry of Health (MS) recommends regular population surveys to identify, through active case detections, infection carriers in the communities and allow for early treatment, through the Schistosomiasis Control Program within the Unified Health System (PCE-SUS), the country's primary health care system. Diagnosis is carried out by parasitological stool examination, preferably by the Kato-Katz method (one sample, two 41.7 mg slides). This method allows visualization and egg count of *Schistosoma mansoni*, also effective in the identification of STH (*Ascaris lumbricoides*, *Trichuris trichiura* and hookworms). *S. mansoni* carriers are treated with a single dose of praziquantel (50–60 mg/kg); whereas STH-positives are administered albendazole or mebendazole. For the endemic areas, the MS recommends biennial periodicity, which may vary according to the epidemiological status of each region, the program development and its impact on the disease [2].

The Rainforest Zone of Pernambuco (ZMP) has long displayed high prevalence rates of infection by *S. mansoni*, despite the successive control campaigns carried out by the MS in its 43 municipalities [3]–[5]. In 1999, with the decentralization of actions from the National Foundation of Health (FUNASA) and the creation of the Priority Action Program of Health Surveillance (PAP-VS), the municipalities became responsible for surveillance and control actions against schistosomiasis through their own health agencies such as the Municipal Coordination of Endemic Diseases (CME). The State Secretary of Health (SES), in turn, was assigned to coordinate these activities and the MS was given the attribution of regulating and supporting state and municipal actions. Information generated on the municipal level is inserted into patient charts, with name, address, gender, date of birth, type of infection and treatment of residents for each of the assisted localities. Such information is sent to the respective Regional Management of Health (GERES) that compile the data and send them to SES for analysis, which is then sent to the Secretary of Health Surveillance (SVS).

**Figure 1:**
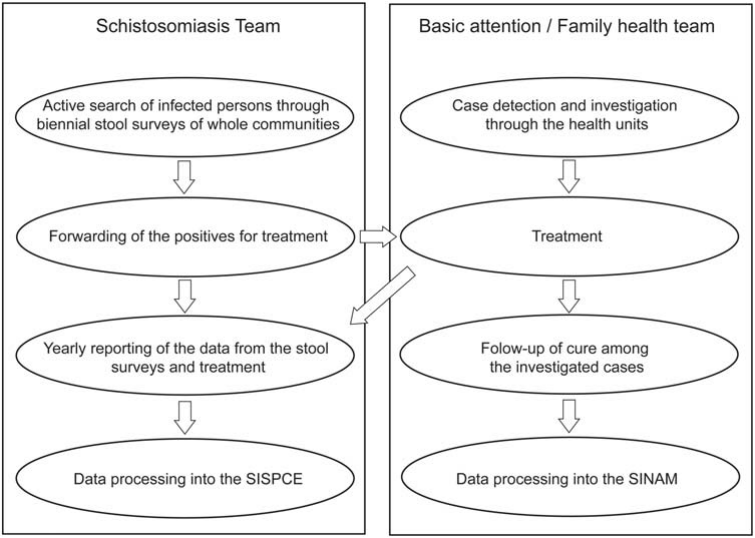
Schistosomiasis Control Program. Flowchart of the activities of the Schistosomiasis Control Program within the Unified Health System (PCE-SUS) at the municipal level.

For endemic areas such as the ZMP the SVS/MS presently recommends that PCE-SUS activities involve two main steps: (i) biennial stool surveys of whole populations at the locality level carried out by a specially formed schistosomiasis team and (ii) treatment of at least 80% of the positives through the local health units. The schistosomiasis team (one leader, one microscopist and at least two field agents) is trained by the SES for distributing and collecting stool vials, preparing and reading Kato-Katz slides as well as forwarding the positives for treatment against schistosomiasis and/or STH under medical supervision at the local health units. Most local health units has Basic Attention-Family Health (AB-SF) teams, each having at least a physician, a nurse and a small number of health attendants and/or house visitors in charge of providing primary health care for up to one thousand families. The SVS recommends a single oral dose of praziquantel (60 mg/kg for children of 2–15 years; 50 mg/kg for older children and adults) against schistosomiasis and a single oral dose of 400 mg albendazole or 500 mg mebendazole against STH via medical prescription [2]. At the doctor's discretion, the patient may take the medication at the health unit or at home. However, the health units must inform the schistosomiasis team of the number of tablets given to each patient or the reason for non-medication (absence, refusal or contraindication). The schistosomiasis team must keep record of all activities for monitoring purposes and present a yearly progress report to the SES for consolidation and evaluation. All information consolidated by the SES is processed into the Computerized System of the Schistosomiasis Control Program (SISPCE). In the municipalities where the schistosomiasis team is not operational or in the non-endemic areas the AB-SF teams are responsible for case detection and investigation among the patients attended at the local health units. This involves identification of the clinical forms and follow-up of cure at four months after treatment (three Kato-Katz exams) as well as reporting of the cases to the Information System for Notifiable Diseases (SINAN). The data notified through the SINAN are not included in the SISPCE. Figure 1 summarizes the PCE-SUS activities carried out by the municipalities.

It must be pointed out that the SVS/MS recommendation of early, regular detection and treatment of the positives aims to prevent increasing morbidity and transmission among all age-groups. This strategy of preventive chemotherapy contrasts with current World Health Organization (WHO) guidelines of treating high-risk groups without prior diagnosis [6]–[13].

Until 2006, the PCE-SUS recorded 237,978 examinations performed in the ZMP [14], which corresponds to only 19,7% of the overall population of 1,207,324 million inhabitants, as estimated by the Brazilian Institute of Geography and Statistics (IBGE) [15]. Considering the fact that the 2000 census registered 222,002 residents between ages 7 and 14 [15] (18.4% of the population in the ZMP), it can be estimated that at least 166,501 children in the 7–14 age range (75.0% of 222,002) still need to undergo examination (and treated if tested positive) by 2010 to reach the goal of WHA-54.19 for the ZMP. Since it is unlikely that the current PCE-SUS actions will suffice to accomplish such a goal in the most troubled municipalities, it has been suggested to include school-aged children as a target group using the school as an operational base [16].

In the present study secondary demographic and parasitological data from a representative municipality of the ZMP are analyzed to provide evidence that the current, community-based approach to control schistosomiasis and STH is unlikely to meet the WHA-54.19 minimum goal, and to reinforce the recommendation that school-based control actions are also needed.

## Methods

### Ethics Statement

The present study was approved by the Oswaldo Cruz Foundation Research Ethics Committee (CEP/Fiocruz) in 16/07/2006, protocol n° 300/05 entitled “Evaluation of the schistosomiasis control actions in the endemic area of Pernambuco within the Unified Health System”.

### Study Area

The municipality of Chã de Alegria was chosen for the present analysis because of the following: (i) its physiographic, climatic and ecological features are typical of the ZMP [5], (ii) its demographic and socio-economical indicators are similar to those of that zone as a whole (Table 1), and (iii) its coverage by the PCE-SUS provides minimally reliable information for data analysis [4].

**Table 1:** Historical prevalence of schistosomiasis and demographic and socio-economic indicators estimated for 2006 in the Rainforest Zone of Pernambuco (ZMP) and the municipility of Chã de Alegria.

Indicators	ZMP	Chã de Alegria
Estimated population	1,207,324	15,288
% prevalence of schistosomiasis 1977–1995	24.1	35.1
% of school-aged children	18.4	19.5
% of enrolled school-aged children	84.6	67.7
% of households without piped-water supply	46.0	54.9
% of households without sewage network	75.4	95.0
Average family earnings (as a proportion of the minimum national wage)	1.6	1.4

### Data Sources

The demographic data were obtained from the Locations Information System of the Unified Health System (SISLOC-SUS), IBGE and the State Secretary of Education (SEE). The parasitological data came from three distinct sources, as follows:

- The summaries of diagnosis and treatment from 2002 to 2006, compiled by the SES of Pernambuco (SES/PE) were used to obtain population data on examination coverage, treatment and prevalence variation of infection in the localities over this period. The compiled data were checked with the source documents from the Municipal Coordination of Endemic Diseases Diseases (CME).
- The CME reports related to the population survey which occurred between September 2003 and May 2004, were used to obtain results from individual examinations at all age levels for each locality.
- The reports from the survey carried out approximately one year later by the Schistosomiasis Reference Service of the Oswaldo Cruz Foundation (SRE/Fiocruz) [17], yielded data from individual examinations in the 7–14 age range.

### Data Management and Analysis

The following data were tabulated by information source, year and locality: number of residents, number of examined, percentage for *S. mansoni* positives among those who were examined and percentage for positives who were treated. For the present analysis, the reliability of prevalence estimates for the localities of the municipality produced by the population surveys was assessed according to criteria recommended by Naing et al. [18] to determine sample size in finite populations. The estimates regarded as reliable were the ones based on a sufficient number of examinations so as to detect the inferior limit of the moderate prevalence level (10%), with a degree of confidence of 95% and accuracy of five percentage points (±5%).

Regarding the school survey, the SRE/Fiocruz had randomly selected eight schools in the municipality, examining in each one of them, at least, 30 students in third and fourth grade classes of elementary school, as advised by Montresor et al. [7],[19]. For the present analysis, significant differences in the proportion of positives in the 7–14 age range between the 2003–2004 population survey (CME) and the 2004 school survey (SRE/Fiocruz) were evaluated using McNemar's test [20].

## Results

### Population Data

The municipality of Chã de Alegria (IBGE code 2604403) was subjected to successive population surveys carried out by the MS, with the following prevalence rates: 33.7% (1977), 28.0% (1979), 39.7% (1982), 41.4% (1984), 44.2% (1992) and 23.5% (1995). This municipality covers an area of 57.9 km^2^ in the ZMP and is 73 km from the state capital, Recife. The major economic activity is agro-industry, with sugarcane, coconut, manioc, sweet potato, banana and mango as their main products [21]. According to the IBGE [15], in 2000 there were 11,102 residents; 2,165 (19.5%) of them school-age children (ages 7–14 yrs). For 2006, the SISLOC-SUS indicates a total of 15,288 residents, with an estimated 2,981 children in the 7–14 age range. According to the SEE, the percentage of the total school-aged population from Chã de Alegria who did not enroll at school in 2006 was 32.7%, whereas the percentage of enrolled school-children who abandoned school (evasion rate) in 2006 was 13.4%. The 2,007 children enrolled at school in 2006 correspond to 89.8% of the minimum goal of the WHA-54.19, which estimated 2,236 children (75% of 2,981).

### Coverage

Table 2 shows a summary of diagnosis and treatment activities for each locality, compiled by SES/PE for the years 2002–2006. Over this period there are records of examinations in all 21 existing localities. Nevertheless, only 11 were surveyed three or more times. A total of 9,838 examinations were done in this period, which corresponds to 64.3% of the population. In the years 2002–2003, 6,861 examinations were performed, 673 (9.8%) were tested positive for *S. mansoni*; 519 (77.1%) people were treated with praziquantel. From 2004 to 2006, there were 2,977 examinations, with 396 (13.3%) positives; from these 396 individuals only 180 (45.4%) received medical treatment.

**Table 2:** Results from stool survey and treatment of the positives in the municipality of Chã de Alegria as compiled yearly by the State Secretary of Health of Pernambuco and made available at the SISPCE [14].

Localities	Res	2002			2003			2004			2005			2006		
		Exa	%Pos	%Tre	Exa	%Pos	%Tre	Exa	%Pos	%Tre	Exa	%Pos	%Tre	Exa	%Pos	%Tre
Alvorada	600	137	7.3	100	-			-			85	5.9*	-	-		
Aratangi	4	4	0.0*	-	-			-			5	0.0*	-	-		
Bela Vista	10	-			-			-			3	0.0*	-	2	0.0*	-
Boa Fé	260	134	22.4	80.0	54	5.6*	100	-			-			-		
Boa Vista	44	8	0.0*	-	-			-			23	21.7*	60.0	22	4.5*	100
Bom Jesus. sítio	78	32	9.4*	66.7	24	16.7*	75.0	-			14	7.1*	-	-		
Bom Jesus. vila	346	285	19.3	76.4	248	7.3	94.4	-			202	6.4	69.2	-		
Brasil	484	267	24.3	75.4	84	8.3*	0.0	59	6.8*	25.0	151	13.9	57.1	-		
Canavieira	68	23	47.8*	81.8	20	45.0*	77.8	-			23	21.7*	100	-		
Chã de Aldeia	268	135	0.0	-	113	4.4	100	-			-			176	6.8	83.3
Chã de Alegria	11,542	3,874	6.0	77.3	0			7	0.0*	-	714	21.3	26.3	705	11.9	72.6
Chã de Anil	152	102	9.8	90.0	53	0.0*	-	-			-			-		
Contendas	72	31	0.0*	-	0			0			38	7.9*	0.0	41	2.4*	100
Lagoinha	436	290	17.2	80.0	201	13.9	78.6	52	5.8*	100	0			327	11.6	34.2
Palheitas	252	118	30.5	77.8	122	17.2	66.7	-			105	11.4	16.7	78	19.2*	60.0
Paroés	200	147	5.4	100	89	3.4	100	-			-			-		
Portões	28	1	0.0*	-	-			-			-			20	5.0*	100
Sítio	164	52	42.3*	86.4	80	13.8	63.6	-			-			-		
Souto	196	46	21.7*	90.0	34	20.6*	28.6	-			46	17.4*	-	-		
Timbó	134	36	36.1*	84.6	-			-			-			55	12.7*	100
Timbó dos Negros	28	16	25.0*	100	-			-			24	25.0*	50.0	-		
Total	15,288	5,739	9.7	78.2	1,122	10.3	71.5	118	5.9	57.1	1,433	16.1	32.0	1,426	11.1	64.8

Res: number of residents in 2006 according to the Locations Information System of the Unified Health System; Exa: number of examined persons; %Pos: percentage of positives for *Schistosoma mansoni*; %Tre: percentage of treated for *S. mansoni*. *: Estimates not reliable to detect 10% of prevalence with 95% of confidence and ±5 percentage points in finite populations [18].

### Prevalence of Infection

As for prevalence levels, five of the working localities (Aratangi, Bela Vista, Boa Vista, Portões and Timbó dos Negros) have populations inferior in number to the minimum necessary to yield reliable estimates. Four other localities (Sítio Bom Jesus, Canavieira, Contendas and Souto) did not provide a sufficient number of examinations to ensure reliability of estimates over the period (Table 2). Apparently, there was no significant prevalence variation in the municipality. The rate rose from 9.7% in 2002 to 11.1% in 2006. However, in Vila Bom Jesus 285 (82.4%) out of 346 residents were examined in 2002, with 55 (19.3%) tested positive; in the following year, 248 examinations were performed (71.7% of the total number of residents), with 18 (7.3%) tested positive.

Table 3 displays the results of the population survey carried out by CME, as well as the data from the school survey by SRE/Fiocruz. In the population survey, 1,766 (11.5%) out of 15,288 residents in the municipality were examined, covering 13 of the 21 localities. Considering the number of individuals tested, the percentage of positives was 11.7%; however, there were no minimally reliable estimates for four localities surveyed by CME. In the school survey, eight elementary schools were sampled: four, at the municipality headquarters, and four others in different rural localities. From the total number of students examined, 20.5% were tested positive.

**Table 3:** Parasitological results from the population survey carried out by the Municipal Coordination of Endemic Diseases Diseases (CME) of Chã de Alegria in 2003–2004 and from the school survey by the Schistosomiasis Reference Service of the Oswaldo Cruz Foundation (SRE/Fiocruz) one year later.

Localities	CME			SER/Fiocruz			
	Exa	Pos	%	Schools	Exa	Pos	%
Boa Fé	49	3	6.1*	-	-	-	-
Bom Jesus. sítio	24	4	16.6*	-	-	-	-
Bom Jesus. vila	246	18	7.3	J.C. Petribu	46	6	13.0
Brasil	255	43	16.9	-	-	-	-
Canavieira	20	9	45.0*	-	-	-	-
Chã de Aldeia	163	10	6.1	Dr A. Jurema	44	6	13.6
Chã de Alegria (town center)	304	40	13.1	Pres. Costa e Silva	38	1	2.6
				A.P. Albuquerque	48	19	39.6
				J.C. Ferraz Filho	34	4	11.8
				J.C. da Silva	42	9	21.4
				Total	162	33	20.4
Chã de Anil	87	7	8.0	-	-	-	-
Lagoinha	250	27	10.8	-	-	-	-
Palheitas	123	19	15.4	M.J. Massena	50	20	40.0
Paroés	107	4	3.7	C.C. de Morais	39	5	12.8
Sítio	35	6	17.1*	-	-	-	-
Souto	103	17	16.5	-		-	-
TOTAL	1,766	207	11.7		341	70	20.5

Exa: number of examined persons; Pos: number of positives for *S. mansoni*. *: Estimates not reliable to detect 10% of prevalence with 95% of confidence and ±5 percentage points in finite populations [18].

From 1,766 individuals examined in the CME survey, 570 were children ages 7–14. From these children, 105 were also counted in the SRE/Fiocruz school survey, held 11 months later. This repeated contact provided individual information about changes in the infection status during this interval (Table 4). In all, 76 children remained negative and eight continued to test positive, whereas 14 became positives and 7 became negatives. The application of the McNemar test indicated, however, that there was no significant change (χ^2^ = 2.333; gl = 1; p>0.05). It is interesting to note that in the four rural localities where the school survey was done, adhesion to the treatment provided by CME, in 2003, was 90.3% on average (see Table 2).

**Table 4:** Status of infection by *S. mansoni* among school-children (7–14 yrs) examined both at the population survey carried by the Municipal Coordination of Endemic Diseases Diseases (CME) of Chã de Alegria in 2003–2004 and in the school survey by the Schistosomiasis Reference Service of Oswaldo Cruz Foundation (SRE/Fiocruz) one year later.

Schools	Status	Neg SRE	Pos SRE	Total
J.C. Petribu	Neg CME	24	3	28
	Pos CME	1	0	
Dr A. Jurema	Neg CME	12	2	17
	Pos CME	2	1	
Pres. Costa e Silva	Neg CME	2	0	3
	Pos CME	1	0	
A.P. Albuquerque	Neg CME	8	0	9
	Pos CME	0	1	
J.C. Ferraz Filho	Neg CME	7	1	8
	Pos CME	0	0	
J.C. da Silva	Neg CME	8	0	10
	Pos CME	0	2	
M.J. Massena	Neg CME	10	6	21
	Pos CME	3	2	
C.C. de Morais	Neg CME	5	2	9
	Pos CME	0	2	
Total	Neg CME	76	14	105
	Pos CME	7	8	

Neg: negatives; Pos: positives.

## Discussion

### Risk Groups

Available data on the current development of population surveys in ZMP make it clear that it is not likely that the PCE-SUS will attain the goal of WHA-54.19 in this important endemic area [4],[16]. The unsatisfactory performance may be due to lack of human and material resources, necessary to the fulfillment of the targets agreed upon by the municipalities in the PAP-VS. At present, the MS recommends actions directed to the population as a whole. In the municipalities where population coverage was unviable in the short run, actions may be focused on risk groups, like school-age children, as anticipated in the National Politics of Basic Health Care Attention (PNAB) [22].

### Treatment Impact

In Chã de Alegria, the number of examinations performed in the years 2002–2003 was 43.4% higher than in the years 2004–2006. As a result, the SES/PE data for this triennium do not allow for an adequate evaluation of treatment impact on the municipality as a whole, since only the capital town presented minimally reliable estimates before and after treatment. However, in Vila Bom Jesus there is evidence of some reduction in the proportion of positives between 2002 and 2003. According to the SES reports, this village was the only locality where two consecutive surveys had wide coverage of exams (82.4% and 71.7%, respectively) as well as treatments (76.4% and 94.4%, respectively). Considering that most residents participated in both surveys and that most positives were treated, the fall from 19.3% to 7.3% in the proportion of positives between the two surveys may reflect a real impact of the treatment on the infection as no other control measure was implemented in the interval between the two surveys.

### Treatment Compliance

The unsatisfactory treatment coverage (45.4%) in the last triennium may be due to the recent SES/PE requirement that medication be prescribed under strict medical supervision, which generally demands from the patient an appointment at a health care center. This implies the cost of time spent while commuting and waiting, which discourages the treatment-seeking adhesion of the patient to the treatment. In addition, many doctors and nurses from primary health care units are unaware of the recent WHO recommendations for the use of praziquantel in clinical practice, which may hinder them to treat patients resulting in unsatisfactory treatment coverage. This has been dealt with through regular seminars carried out by the SES/PE to update their knowledge [4].

### Reliability of Prevalence Estimates

It is worth noting that the PCE-SUS parasitological surveys do not aim to produce prevalence estimates in the localities under consideration, but rather identify carriers of infection for treatment. If biennial surveys of whole endemic communities and treatment of the positives are carried out as recommended by the MS, then reliable information on the prevalence of infection and the treatment impact can be gathered from the SISPCE. Unfortunately, the number of examinations agreed upon by the ZMP municipalities every year through the PAP-VS only covers a small part of the population at risk. As a result, the surveys usually obey to operational and political priorities, which may compromise the reliability of the data [5]. It is hoped that as the PCE-SUS evolves, making available more resources from the federal level to the municipalities, both population coverage and data retrieval from different epidemiological settings will improve satisfactorily. In the meantime, it would be advisable that the municipalities planned their surveys taking into account sampling criteria that prevent accuracy and validity problems and that allow the obtainment of reliable prevalence estimates.

Another problem that hinders the correct evaluations of PCE-SUS actions is the discrepancy of available data at municipal and state levels [5],[16]. In Chã de Alegria, the CME report of the 2003–2004 survey indicates 1,766 examinations, while the SES summary for the same two years presents a total of 1,240 examinations. This demonstrates a loss of 526 (29.8%) examinations between the municipal and state levels. Such a problem may be prevented with the correction of inaccuracies and inconsistencies in the information flow [23].

### Follow-Up

A further issue is the lack of follow-up of individuals in successive surveys, hampering impact evaluation of control actions. The ideal would be that conclusions about prevalence variation at locality or municipality levels were made on the basis of the results of individuals examined before and after control actions. In Chã de Alegria, the parasitological information of a school survey made about a year after the 2003–2004 population survey allowed the evaluation of the statistical significance of changes in infection status among children who were examined on both occasions. The absence of significant difference between the surveys may be mainly due to high re-infection rates historically recorded in ZMP [24],[25], once the adhesion rate to treatment in the population survey was superior to the 80% level recommended by MS.

### School-Based Interventions

A short-term alternative scheme, capable of abiding by the Resolution 54.19, adjusting WHO [26] and MS [2] recommendations for diagnosis and treatment in the most troubled municipalities, would be to make diagnosis and treatment using elementary schools as the operational base. In Chã de Alegria, ample coverage of elementary schools would be enough to reach and exceed the minimum goal of WHA-54.19 for 2010, once it is complemented by active search of school-aged children who live in the surrounding area but do not attend school. It is important to point out that the estimated number of children to reach this goal (2,236) lies within the annual average of examinations agreed upon by the municipality through PAP-VS.

Surveillance and control strategies in the medium and long terms should take into consideration the local epidemiological characteristics and the availability of material and human resources. For example, in the state of São Paulo, where schistosomiasis constituted a public health problem until the 1970's, current epidemiological surveillance actions encompass: (i) compulsory notification of cases identified in laboratories and healthcare services; (ii) parasitological surveys of elementary school students in the priority municipalities followed by treatment of the positives and (iii) investigation of cases and analytical epidemiological evaluation [27]. On the other hand, in states like Pernambuco, where the endemic disease still represents a serious public health problem and local infrastructure conditions hinder the satisfactory implementation of control actions, efforts must contemplate, at least, more vulnerable groups, such as school-aged children [16].

Periodic surveys conducted in schools have the following advantages [12]: (i) schools are accessible and receptive; (ii) the highest prevalence rates of infection are found among school-age children; (iii) data collected in this age range may be used to evaluate not only if schistosomiasis threatens the health of school-age children, but also if there is need for intervention in the community as a whole; (iv) children in intermediate grades (generally between ages 9–12) allow for the accompaniment of treatment impact over one or two years, before they leave school.

An important limitation of the school-based approach is that a significant proportion of the school-aged children may not attend school. In order to overcome this problem, the evaded school children may be reached at their homes from the personal information provided by the enrolment school records. The non-enrolled school-aged children may be reached with the help of local organizations, community leaders, teachers and students, and invited to the school on special days to participate in health education activities and be screened for treatment [7]. Outreach to non-enrolled school-aged children can be improved with the strategy proposed by Massara et al. [28] and put in practice by Enk et al. [29] using stool surveys among school-children as an indicator for the identification of positives in the family circle. According to these authors, the school has proved to be a privileged space in the community to approach issues of prevention and disease control. This strategy may generate large-scale actions of health promotion directed not only to school-children, but family members as well.

### Integrated Control

The adoption of schools as the operational base for diagnostic and treatment actions do not discard the need for other control measures outside the school environment. Thus, diagnosis and treatment for schistosomiasis and STH should be available to all patients who seek attendance at the local health units, particularly the most vulnerable groups. In addition, auxiliary measures such as safe-water supply, sanitation and snail control, as well as community mobilization and strategies of information, education and communication should be applied in accordance to the reality of each area.

The proposal for the delivery of diagnosis and treatment using schools as the operational base is inserted in the basic strategy for the control of schistosomiasis and STH in endemic areas established by the PAP-VS instructional guidelines, which consist of intensive, systematic and regular use of stool surveys to identify infected individuals and promote early treatment. Furthermore, it serves the PNAB [22], which fosters the development of actions focused on risk groups in the working process of the AB-SF teams. However, it must be made clear that the involvement of elementary schools with control actions depends on the decision of each municipality, supported by the Municipal Health Council (CMS), especially in the provision of resources to the attainment of agreed goals.

### Experiences of Other Countries

The Brazilian program based on municipal government sovereignty and implementation is in contrast with other schistosomiasis control programs, where the model is implemented directly from the national level to the regional or local level, as follows:

In Egypt control measures are implemented by the national level through the primary health care system, based on selective population chemotherapy and mass chemotherapy for rural school-children and populations in areas of high prevalence and risk. Experience suggests that the control of schistosomiasis is optimal when specific control tasks are carried out within the primary health care system. However, there are hot spots where schistosomiasis transmission still occurs, which require a good surveillance system [30].

In Uganda, the national government is responsible for the planning and implementation of the control program. Annual mass treatment is provided to whole populations where prevalence exceeds 50%. In communities where the prevalence is 20% to 50%, only school-aged children receive annual mass treatment. In communities where prevalence is below 20% health facility based treatment is encouraged and health education intensified. Treatment in schools is carried out by teachers and in communities by community drug distributors, who are selected by the concerned communities and trained by the district trainers. All districts carry out annual deworming on special treatment days. Drug distribution in schools is rated as excellent and community-directed treatment is considered a feasible health approach for mass drug distribution in poor remote communities. The main limitations are that (i) sustained regular mass deworming is hampered by high cost of delivering treatment and (ii) a large number of children are not at school. It is understood that greater effort must be made to reach non-enrolled children to meet the WHA resolution 54.19 minimum goal and to integrate deworming into already existing and successful disease control campaigns [30]–[32].

In Nigeria the Ministry of Health makes decisions on schistosomiasis haematobium treatment based on assessments at the village level. Mobile teams test urine for blood in random samples of 30 children (aged 10–14 years) drawn from one randomly selected school. Guidelines require stratification of villages into three groups according to urine blood prevalence: those who do not qualify for praziquantel mass treatment (<20%); mass treatment of school-aged children (20–49%); and community-wide treatment (>50%). Since 1999, drug distribution is conducted by community-based volunteers. A dramatic decrease in the prevalence of blood in the urine of schoolchildren was reported within three years of instituting the treatment program. However, the cost of praziquantel still hinders the coverage of treatment [33],[34].

In Cambodia the Ministry of Health carries out control activities consisting mainly of yearly administration of praziquantel (40 mg/kg) to the entire population, except for children under 2 years of age and pregnant women. The monitoring surveys have been conducted in school aged children. No new cases of severe morbidity due to schistosomiasis have been reported in the past four years in the health facilities in the area. However it is recognized that if the drug pressure is not maintained the parasite could easily return to original levels due to poor sanitation standards [35].

In China, the central and local governments have sustained commitment to schistosomiasis control through mass chemotherapy once a year targeted at people aged 6–60 years in highly endemic areas of *Schistosoma japonicum* (infection rate ≥15%). Chemotherapy has been extended to domestic animals in combination with snail control by chemical mollusciciding. This program has been particularly successful in interrupting transmission in five provinces. However, there is concern regarding low compliance after repeated praziquantel administration, cost of treatment and the potential risk of drug resistance. It is recommended that, in the long-term, control efforts be absorbed into more horizontal “sector-wide” approaches [30],[36],[37].

In the Philippines the national schistosomiasis control program is based on selective mass chemotherapy. Stool exam of school-children is used as indicator of mass treatment in the community if the prevalence is 10% and above. It is recognized that the program should be properly assessed and revised to include environmental sanitation and snail control, as well as better surveillance measures [38].

### Conclusion

It is clear that the lessons learned from the specific context of the present work may not be fully transferable to the experiences of other countries. However, considering other likely scenarios from Africa and Asia, it is hoped that the present proposal of adjusting the program to cover school-aged children may serve as an option for consideration in regions which experience similar difficulties to meet the WHA resolution 54.19.

## References

[1] World Health Organization (2001). World Health Assembly Resolution 54.19 Schistosomiasis and soil-transmitted helminth infections.. http://www.who.int/wormcontrol/about_us/en/ca54r19.pdf.

[2] Secretaria de Vigilância em Saúde (2005). Esquistossomose Mansônica. In: Ministério da Saúde. Guia de vigilância epidemiológica.

[3] Favre TC, Pieri OS, Barbosa CS, Beck L (2001). Avaliação das ações de controle da esquistossomose implementadas entre 1977 e 1996 na área endêmica de Pernambuco, Brasil.. Rev Soc Bras Med Trop.

[4] Favre TC, Ximenes RAA, Galvão AF, Pereira APB, Wanderley TN (2006). Attaining the minimum target of resolution WHA 54.19 for schistosomiasis control in the Rainforest Zone of the state of Pernambuco, Northeastern Brazil.. Mem Inst Oswaldo Cruz.

[5] Favre TC, Ximenes RAA, Galvão AF, Pereira APB, Wanderley TN (2006). Reliability of current estimates of schistosomiasis prevalence in the Rainforest Zone of the state of Pernambuco, Northeastern Brazil.. Mem Inst Oswaldo Cruz.

[6] Allen HE, Crompton WT, de Silva N, LoVerde PT, Olds GR (2002). New policies for using anthelmintics in high risks groups.. Trends Parasitol.

[7] Montresor A, Crompton D, Gyorkos TW, Saviolli L (2002). Helminth Control in School-age Children: A Guide for Managers of Control Programmes.

[8] World Health Organization (2002). Prevention and control of schistosomiasis and the soil-transmitted helminthiasis. Report of a WHO Expert Committee.

[9] Savioli L, Stansfield S, Bundy DAP (2002). Schistosomiasis and soil-transmitted helminth infections: forging control efforts.. Trans R Soc Trop Med Hyg.

[10] Savioli L, Albonico M, Engels, Montresor A (2004). Progress in the prevention and control of schistosomiasis and soil-transmitted helminthiasis.. Parasitol Internal.

[11] Savioli L, Engels D, Roungou JB, Fenwick A, Endo H (2004). Schistosomiasis control.. Lancet.

[12] Savioli L, Engels D, Endo H (2005). Extending the benefits of deworming for development.. Lancet.

[13] Crompton DWT, Engels D, Montresor A, Neira MP, Savioli L (2003). Action starts now to control disease due to schistosomiasis and soil-transmitted heminthiasis.. Acta Trop.

[14] Ministério da Saúde Secretaria de Vigilância em Saúde. Programa de Controle da Esquistossomose. Departamento de Informática do Sistema Único de Saúde - DATASUS.. http://tabnet.datasus.gov.br/cgi/tabcgi.exe?sinan/pce/cnv/pce.def.

[15] Instituto Brasileiro de Geografia e Estatística Censo demográfico de 2000: resultados.. http://www.ibge.gov.br.

[16] Pieri OS, Favre TC (2007). Incrementando o Programa de Controle da Esquistossomose.. Cad Saúde Pública.

[17] Barbosa CS, Favre TC, Wanderley TN, Callou AC, Pieri OS (2006). Assessment of Schistosomiasis, through school surveys, in the Forest Zone of Pernambuco, Brazil.. Mem Inst Oswaldo Cruz.

[18] Naing L, Winn T, Rusli BN (2006). Practical issues in calculating the sample size for prevalence studies.. Arch Orofac Sci.

[19] Montresor A, Crompton DWT, Hall A, Bundy DAP, Savioli L (1998). Guidelines for the evaluation of soil-transmitted helminthiasis and schistosomiasis at community level.

[20] Sokal RR, Rohlf FJ (1995). Biometry: the principles and practice of statistics in biological research.

[21] Instituto de Planejamento de Pernambuco (2001). Mesorregião da Mata Pernambucana: Microrregiões da Mata Setentrional, da Mata Meridional e de Vitória de Santo Antão. Monografia Mesorregional.

[22] Ministério da Saúde (2006). Política Nacional de Atenção Básica. Secretaria de Atenção à Saúde. Departamento de Atenção Básica.

[23] Farias LMM, Resendes APC, Sabroza PC, Souza-Santos R (2007). Análise preliminar do Sistema de Informação do Programa de Controle da Esquistossomose no período de 1999 a 2003.. Cad Saúde Pública.

[24] Moza PG, Pieri OS, Barbosa CS, Rey L (1998). Fatores sócio-demográficos e comportamentais relacionados à esquistossomose em uma agrovila da zona canavieira de Pernambuco, Brasil.. Cad Saúde Pública.

[25] Pieri OS, Barbosa CS, Moza PG (1998). Schistosomiasis control based on repeated chemotherapy in a rural village of the sugar-cane zone in Northeast Brazil.. Mem Inst Oswaldo Cruz.

[26] World Health Organization (2006). Preventive chemotherapy in human helminthiasis. Coordinated use of anthelminthic drugs in control interventions: a manual for health professionals and programme managers.

[27] Centro de Vigilância Epidemiológica Prof. Alexandre Vranjac (2006). Vigilância Epidemiológica e Controle da Esquistossomose: Normas e Instruções. São Paulo.. http://www.cve.saude.sp.gov.br/doc_tec/hidrica/doc/manu_esqui.pdf.

[28] Massara CL, Peixoto SV, Enk MJ, Barros HS, Carvalho OS (2006). Evaluation of an improved approach using residences of schistosomiasis-positive school children to identify carriers in an area of low endemicity.. Am J Trop Med Hyg.

[29] Enk MJ, Lima ACL, Massara CL, Coelho PMZ, Schall VT (2008). A combined strategy to improve the control of Schistosoma mansoni in areas of low prevalence in Brazil.. Am J Trop Med Hyg.

[30] World Health Organization (2005). Report of the Scientific Working Group meeting on Schistosomiasis. Special Programme for Research & Training in Tropical Diseases (TDR).

[31] Kabatereine NB, Fleming FM, Nyandindi U, Mwanza JCL, Blair L (2006). The control of schistosomiasis and soil-transmitted helminths in East Africa.. Trends Parasitol.

[32] Kabatereine NB, Tukahebwa E, Kazibwe F, Namwangye H, Zaramba S (2006). Progress towards countrywide control of schistosomiasis and soil-transmitted helminthiasis in Uganda.. Trans R Soc Trop Med Hyg.

[33] Richards FO, Eigege A, Miri ES, Jinadu MY, Hopkins D (2006). Integration of mass drug administration programmes in Nigeria: the challenge of schistosomiasis.. Bull World Health Organ.

[34] The Carter Center Schistosomiasis Control Program.. http://htpp://www.cartercenter.org/health/Schistosomiasis/index.html.

[35] Sinuon M, Tsuyuoka R, Socheat D, Odermatt P, Ohmae H (2007). Control of Schistosomiasis mekongi in Cambodia: results of eight years of control activities in the two endemic provinces.. Trans R Soc Trop Med Hyg.

[36] Jia-Gang G, Chun-Li C, Guang-Han H, Han L, Gong L (2005). The role of “passive chemotherapy” plus health education for schistosomiasis control in China during maintenance and consolidation phase.. Acta Tropica.

[37] Xiao-Nong Z, Li-Ying W, Ming-Gang C, Tian-Ping W, Jia-Gang G (2005). An economic evaluation of the national schistosomiasis control programme in China from 1992 to 2000.. Acta Tropica.

[38] Blas BL, Rosales MI, Lipayon IL, Yasuraoka K, Matsuda H (2004). The schistosomiasis problem in the Philippines: a review.. Parasitol Int.

